# SS18 Together with Animal-Specific Factors Defines Human BAF-Type SWI/SNF Complexes

**DOI:** 10.1371/journal.pone.0033834

**Published:** 2012-03-19

**Authors:** Evelien Middeljans, Xi Wan, Pascal W. Jansen, Vikram Sharma, Hendrik G. Stunnenberg, Colin Logie

**Affiliations:** Department of Molecular Biology, Nijmegen Centre for Molecular Life Sciences, Radboud University, Nijmegen, The Netherlands; Oregon State University, United States of America

## Abstract

**Background:**

Nucleosome translocation along DNA is catalyzed by eukaryotic SNF2-type ATPases. One class of SNF2-ATPases is distinguished by the presence of a C-terminal bromodomain and is conserved from yeast to man and plants. This class of SNF2 enzymes forms rather large protein complexes that are collectively called SWI/SNF complexes. They are involved in transcription and DNA repair. Two broad types of SWI/SNF complexes have been reported in the literature; PBAF and BAF. These are distinguished by the inclusion or not of polybromo and several ARID subunits. Here we investigated human SS18, a protein that is conserved in plants and animals. SS18 is a putative SWI/SNF subunit which has been implicated in the etiology of synovial sarcomas by virtue of being a target for oncogenic chromosomal translocations that underlie synovial sarcomas.

**Methodology/Principal Findings:**

We pursued a proteomic approach whereby the SS18 open reading frame was fused to a tandem affinity purification tag and expressed in amenable human cells. The fusion permitted efficient and exclusive purification of so-called BAF-type SWI/SNF complexes which bear ARID1A/BAF250a or ARID1B/BAF250b subunits. This demonstrates that SS18 is a BAF subtype-specific SWI/SNF complex subunit. The same result was obtained when using the SS18-SSX1 oncogenic translocation product. Furthermore, SS18L1, DPF1, DPF2, DPF3, BRD9, BCL7A, BCL7B and BCL7C were identified. ‘Complex walking’ showed that they all co-purify with each other, defining human BAF-type complexes. By contrast,we demonstrate that human PHF10 is part of the PBAF complex, which harbors both ARID2/BAF200 and polybromo/BAF180 subunits, but not SS18 and nor the above BAF-specific subunits.

**Conclusions/Significance:**

SWI/SNF complexes are found in most eukaryotes and in the course of evolution new SWI/SNF subunits appeared. SS18 is found in plants as well as animals. Our results suggest that in both protostome and deuterostome animals, a class of BAF-type SWI/SNF complexes will be found that harbor SS18 or its paralogs, along with ARID1, DPF and BCL7 paralogs. Those BAF complexes are proteomically distinct from the eukaryote-wide PBAF-type SWI/SNF complexes. Finally, our results suggests that the human bromodomain factors BRD7 and BRD9 associate with PBAF and BAF, respectively.

## Introduction

Gene expression programs determine cell identity and response to endocrine stimuli, as has been demonstrated most dramatically by the generation of induced pluripotent stem cells with the Oct4, Sox2, Klf4 and c-Myc transcription factors [1]. Such epigenetic programming involves many nucleosome remodeling activities [2]. Besides covalent nucleosome modifications such as acetylation and methylation, a second type of nucleosome remodelling involves translocation of nucleosomes along chromosomal DNA [3]–[5] as well as the catalysis of alternative nucleosome conformations, and even nucleosome eviction [6]–[8]. These nucleosome transactions are catalyzed by SNF2 type enzymes, a group of ATPases that belongs to the SFII ATPase superfamily that includes many helicases [9]. The present paper is concerned with a subtype of the nucleosome remodeling SNF2 enzymes that are uniquely characterized by a C-terminal bromodomain, represented in yeast by Snf2 and Sth1, in *Drosophila* by brahma and in humans by BRM and BRG1.

The C-terminal bromo domain-bearing SNF2 enzymes are found in so-called SWI/SNF multiprotein complexes and are conserved in most eukaryotes. They are implicated in transcriptional regulation and multiple DNA repair pathways [10]–[25]. These large multi-protein complexes consist of at least 4 evolutionarily conserved core subunits represented in man by SMARCB1 and the SMARCA2/A4, SMARCC1/C2 and SMARCD1/D2/D3 paralogs [26], and a large number of ancillary subunits, some of which define SWI/SNF complex subtypes. Interestingly, SWI/SNF complexes were identified as biochemical factors that dramatically reduce the amount of time required to reprogram mouse embryonic fibroblasts into iPS at the hand of recombinant transcription factors [27], underscoring the importance of SWI/SNF in epigenetic programming processes [28]. Indeed, SWI/SNF has been mapped to some 50,000 human chromosomal sites in one cultured human cell line, demonstrating that this protein complex is a feature of many cis-acting regulatory elements, including DNA replication origins [29].

In mice and humans, at least 20 different SWI/SNF complex subunits have been reported (Table 1) [10], [30]–[43]. ‘Core’ subunits are found in virtually all cellular SWI/SNF complexes, whilst others define SWI/SNF complex subtypes. There are two broad classes of SWI/SNF complexes known; BAF-type SWI/SNF complexes (BRG1/BRM-associated factors) bear either one of ARID1A/BAF250a or ARID1B/BAF250b, whilst PBAF (Polybromo-associated BAF) complexes harbor both ARID2/BAF200 and polybromo/BAF180 subunits [38], [39], [44], [45]. Functionally, ARID1B/BAF250b was shown to be required to maintain ES cell identity [46] whilst ARID1A/BAF250a is required to permit proper ES cell differentiation with retinoic acid [47]. Furthermore, the SMARCC variant BAF170 is expressed less upon ES cell differentiation [43], [48], [49]. Similarly, the switch from one actin related subunit, BAF53A, to its paralog BAF53B appears to play a key role in neuron progenitor differentiation [41], [50]. Tissue specific expression of paralogous subunits has also been reported for the BAF60 variants [32], [51]–[53], as well as for the minor SWI/SNF subunits DPF1 and 3 [41], [42], [50].

**Table 1:** Abundance of purified proteins‡ in each TAP-tag preparation.

Protein	alternative names	polyA mRNA*	TAP	INI1	SS18	SS18SSX1	BCL7A	BCL7C	DPF2	BRD9	PHF10
BRG1	SMARCA4	5.93	0	0.303	5.337	4.440	2.039	2.290	1.662	0.141	0.984
BRM	SMARCA2	1.45	0	0	1.339	0.730	0.116	0.280	0.179	0	0.028
BAF250A	SMARCF1, ARID1A	1.63	0	0.027	5.692	2.079	0.379	1.766	1.485	0.027	0.027
BAF250B	ARID1B, OSA1	2.04	0	0	2.728	1.540	0.179	0.638	0.315	0.028	0
BAF200	ARID2, zipzap	nd	0	0.122	0	0.029	0.029	0.122	0	0	0.884
BAF180	Polybromo-1	4.36	0	0.457	0	0	0	0.248	0	0	1.769
BAF170	SMARCC2	9.25	0	1.532	2.793	2.360	0.438	1.432	1.154	0.084	1.745
BAF155	SMARCC1	6.94	0	2.981	5.813	3.467	1.239	2.043	2.831	0.080	0.468
BAF60A	SMARCD1	4.01	0	0.15	3.037	3.037	0.784	2.054	1.477	0.072	0.630
BAF60B	SMARCD2	7.69	0	0.719	7.161	4.080	1.762	2.875	2.384	0	0.607
BAF60C	SMARCD3	3.02	0	0	1.102	0.346	0	0.346	0.16	0	0
BAF57	SMARCE1	9.97	0	0.957	11.115	5.190	1.154	3.642	3.217	0.957	1.61
BAF53A	ACTL6A, ArpNß	8.37	0.233	0.110	6.305	6.305	2.511	2.511	0.874	0.369	0.52
BAF53B	ACTL6B, ArpNα	1.68	0	0	0	0	0	0	0	0	0
BAF47	SMARCB1, INI1, SNF5	5.81	0	**0.931**	5.449	3.160	1.154	3.160	0.551	0.245	0.823
BAF45A	PHF10	3.08	0	0.086	0	0	0	0	0	0	**2.728**
BAF45B	DPF1	0.50	0	0	0	0.110	0	0.110	0	0	0
BAF45C	DPF3, CERD4	0.26	0	0	0	0	0	0.105	0	0	0
BAF45D	DPF2, REQ, UBID4	2.80	0	0	4.623	1.610	0.101	1.371	**1.873**	0	0
SS18	SYT, SSXT	3.73	0	0	**9.00**	**9.00**	0.78	0	2.16	1.000	0
SS18L1	CREST	9.92	0	0	0	0	0	0.585	0.585	0	0
BCL7A	-	nd	0	0	0.874	1.310	**3.329**	0	0.585	0.233	0
BCL7B	Hom s 3	4.77	0	0	0	0.292	0	0	0	0	0
BCL7C	-	4.18	0	0	0	1.783	0	**11.915**	0	0	0
BRD7	CELTIX-1	nd	0	0.064	0	0	0	0	0	0	0.645
BRD9	MU-RMS-40.8	0.18	0	0	0.186	0.668	0	0.089	0	**4.505**	0
SSX1	-	nd	0	0	0	**1.154**	0	0	0	0	0
GLTSCR1	GSCR1	1.20	0	0	0.619	1.116	0.055	0.708	0	0	0
SRRM2	-	8.15	0	0	0	0	0	0	0	0	0
MYBBP1A	p160	3.72	0	0	0	0	0	0	0	0	0
NONO	NMT55, p54(nrb)	nd	0.066	0	0	0.066	0	0	0	1.966	0.292
NUMA1	-	3.23	0	0	0	0	0	0	0	0.167	0
SFPQ	PSF	11.42	0.199	0	0	0	0	0	0	0.624	0.528
DDX3X	HLP2	12.18	0.116	0	0	0	0	0	0	10.159	0.315
DDX17	p72	11.85	0.058	0	0	0	0	0	0.058	9.578	0.136
RBM14	COAA	7.10	0	0	0	0	0	0	0	4.736	0
DDX5	p68	17.37	0	0	0	0	0	0	0.061	4.223	0.805
actin	actg1	30.37	4.109	1.371	4.109	6.499	2.481	2.831	0.778	7.254	3.437

‡ Protein abundance is represented by the exponentially modified Protein Abundance Index [96].

* mRNA abundance was estimated from probe set fluorescence signal intensities, as recommended by Affymetrix (see Data S2).

Strikingly, multiple SWI/SNF subunits function as tumor suppressors in man and mouse, adding a key medical dimension to SWI/SNF research [24], [54]–[66]. For instance: the INI1^flox/flox^ mouse is the most lethal tumor suppressor mouse model reported to date [55], suggesting a decisive role for SWI/SNF in cell proliferation control. Cell cycle roles for SWI/SNF-type complexes have indeed been documented in human and in model organisms [67]–[78].

Another link to cancer is provided by the SS18-SSX oncofusion proteins [79]. Synovial sarcomas are aggressive soft-tissue tumors accounting for about ten percent of all human soft-tissue sarcomas [80]. Characteristic for synovial sarcomas is the t(X;18)(p11.2;q11.2) translocation which is found in over 95% of all synovial sarcoma cases and results in the fusion of the SS18 (also called Syt) gene on chromosome 18 with one of the highly homologous SSX genes, SSX1, SSX2 or SSX4, on the X chromosome and consequently the expression of SS18-SSX fusion proteins [81]–[84]. These translocation events are believed to be the main molecular basis of this disease [79]. Orthologs of the SS18 protein also exists in plants. They are positive regulator of cell proliferation in lateral organs, such as leaves and flowers and appear to control aspects of cell proliferation together with DNA sequence-specific GRF transcription factors [85], [86]. Mammalian SS18 has been reported to associate with SWI/SNF chromatin remodeling complexes and to interact with BRG1 and BRM proteins [87]–[90]. In order to identify protein interactors of the SS18 and SS18-SSX proteins and to characterize the SS18 and SS18-SSX complexes we exploited a Tandem Affinity Purification (TAP) tagging approach combined with mass spectrometric analysis [91].

We found SS18 to be present in BAF-class human SWI/SNF chromatin remodeling complexes. Purification of SS18-SSX1 revealed that this oncofusion protein resides in the same complexes. Interestingly, we detected several additional putative SWI/SNF interactors [92]–[95]. Complex walking revealed the presence of these proteins in the same BAF SWI/SNF complexes as SS18, refining observations made by others [41], [43]. Overall, we conclude that human SS18 and its paralogue SS18l1/CREST together with; double PHD finger factors (DPF1,-2,-3), the B-cell CLL/lymphoma 7 protein family members (BCL7A, -B, -C) and BRD9 are specific to BAF-class SWI/SNF complexes, whilst BRD7 and PHF10 characterize PBAF complexes. Furthermore, with the exception of BRD7 and BRD9, quantitative mass spectrometry analysis demonstrates that the major proteomic interaction partners of all these factors are SWI/SNF subunits, indicating that they are *bona fide* BAF-type SWI/SNF complex subunits.

## Results

### TAP-tag purification of SWI/SNF complexes

**Figure 1:**
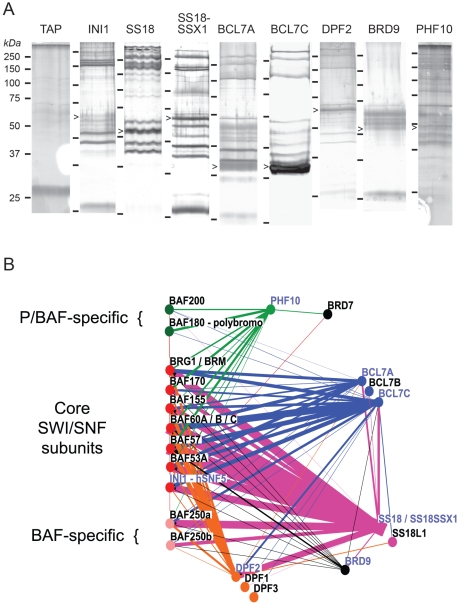
Complex walking. (A) Silver-stained gels of the actual purified protein preparations that were analyzed by mass spectrometry. The respective TAP-tag fusion proteins are designated by black triangles. Size markers (Da) are indicated for every gel. Banding patterns differ because the gels were not all run under the same conditions. (B) Osprey interaction network [131] based on the mass spectrometry results obtained with the material shown in panel A. Blue name labels indicate the TAP-tag fusion employed here. The thickness of the lines reflect purification yield and are proportional to the emPAI values [96] shown on Table 1. The presence of orthologs in yeast and *Drosophila* genomes of the human factors that are displayed is indicated on Table 2.

In order to define the protein complexes harboring known and suspected human SWI/SNF subunits we generated stable human embryonic kidney cell (Hek293) clones transduced with retroviral TAP-tag fusion expression constructs [91]. The following eight TAP-fusions were purified and analyzed by mass spectrometric analysis; INI1, SS18 and its oncogenic fusion product SS18-SSX1, BCL7A and BCL7C, DPF2, PHF10 and BRD9 (Figure 1A). The proteomic data we have collected (Table 1, Data S1) is schematized in Figure 1B, where the thickness of the edges reflect co-purification efficiency [96].

### INI1

INI1 is a core subunit of SWI/SNF complexes that is also known as hSNF5, SMARCB1 or BAF47. In our hands the yield of SWI/SNF complexes obtained with INI1^TAP^ has consistently been comparatively low. For instance, in most INI1^TAP^ preparations we detect BRG1 but not BRM, and ARID1A but not ARID1B (Table 1). This is consistent with BRG1 and ARID1A mRNA levels being 2–3 times that of their respective paralogs in Hek293 cells (Table 1, Data S2), a fact that is also reflected in the yields of these paralogous subunits in all the purifications (Table 1). Another indication that the INI^TAP^ construct is not amenable to very high yield SWI/SNF purifications is that of all the proteins we employed here to purify SWI/SNF complexes, only two, PHF10 and BRD7, are detected by INI1^TAP^, whilst INI1 was detected in all the reciprocal purifications (Table 1).

In keeping with a role as a core SWI/SNF subunit, INI1^TAP^ purifications harbored both PBAF and BAF-specific SWI/SNF subunits (Table 1, Table 2). The comparatively higher yield of the PBAF-specific subunits ARID2 and polybromo versus the BAF-specific ARID1A and ARID1B suggests that INI1-bearing PBAF complexes are more preponderant than INI1-bearing BAF complexes in Hek293 cells, in line with the higher expression level of the PBAF-specific polybromo subunit (Table 1).

**Table 2:** Orthologs of known SWI/SNF complex subunits in human, fly and yeast.

Human Protein	Alternative human names	Human complex	*Drosophila melanogaster*	Fly complex	*Saccharomyces cerevisiae* SWI/SNF	*Saccharomyces cerevisiae RSC * [*134*]
BRG1	SMARCA4	Core	brahma/CG5942	Core	SNF2	STH1
BRM	SMARCA2	Core	brahma/CG5942		SNF2	STH1
BAF250A	SMARCF1, ARID1A	BAF	OSA/eyelid/CG7467	BAP	SWI1	-
BAF250B	ARID1B, OSA1	BAF	OSA/eyelid/CG7467		SWI1	-
BAF200	ARID2, zipzap	PBAF	BAP170/CG3274	PBAP	-	-
BAF180	Polybromo-1	PBAF	polybromo/BAP180/CG11375	PBAP	-	RSC1, RSC2, RSC4
BAF170	SMARCC2	Core	moira/BAP155/CG18740	Core	SWI3	RSC8
BAF155	SMARCC1	Core	moira/BAP155/CG18740		SWI3	RSC8
BAF60A	SMARCD1	Core	BAP60/CG4303	Core	SWP73	RSC6
BAF60B	SMARCD2	Core	BAP60/CG4303		SWP73	RSC6
BAF60C	SMARCD3	Core	BAP60/CG4303		SWP73	RSC6
BAF57	SMARCE1	Core	dalao/BAP111/CG7055	Core	-	-
BAF53A	ACTL6A, ArpNb	Core	BAP55/CG6546	Core	ARP7 & ARP9	ARP7 & ARP9
BAF53B	ACTL6B, ArpNa	Core	?		ARP7 & ARP9	ARP7 & ARP9
BAF47	SMARCB1, INI1, SNF5	Core	SNR1/CG1064	Core	SNF5	SFH1
BAF45A	PHF10	PBAF	e(y)3/SAYP/CG12238	PBAP	-	-
BAF45B	DPF1	BAF	d4/CG2682	?	-	-
BAF45C	DPF3, CERD4	BAF	d4/CG2682		-	-
BAF45D	DPF2, REQ, UBID4	BAF	d4/CG2682		-	-
SS18	SYT, SSXT	BAF	CG10555	?	-	-
SS18L1	CREST	BAF	CG10555		-	-
BCL7A	-	BAF	BCL7-like/CG17252	?	-	-
BCL7B	Hom s 3	BAF	BCL7-like/CG17252		-	-
BCL7C	-	BAF	BCL7-like/CG17252		-	-
BRD7	CELTIX-1	PBAF	CG7154	?	-	-
BRD9	MU-RMS-40.8	BAF	CG7154		-	-
actin	actg1		actin		actin	actin
					RTT102	RTT102
					SWP82	NPL6
						HTL1
						LDB7
						RSC3
						RSC30
						RSC58
						RSC9
					SNF6	

### SS18 and the oncogenic SS18-SSX fusions are BAF subunits

SS18^TAP^ purifications yielded high levels of SWI/SNF (Table 1, Figure 1). All known core subunits were found, consistent with previous work [87], [97]. Since both ARID1A and ARID1B but no ARID2 nor polybromo peptides were found, SS18 appears to be specific to both the ARID1A and ARID1B-bearing BAF-class variants of SWI/SNF (Table 1). Furthermore, several other potential SS18 interactors were identified, including GLioma Tumor Suppressor Candidate Region gene 1 protein (GLTSCR1), zinc finger protein ubi-d4 (DPF2), B-cell CLL/lymphoma 7A (BCL7A) and bromodomain containing protein 9 (BRD9) (Table 1, Figure 1B).

Because the chromosomal translocation t(X;18)(p11.2;q11.2) results in production of the oncogenic SS18-SSX1 protein fusion it was of interest to compare the proteomic environments of SS18 and the SS18 oncofusions. Essentially, purification of SS18-SSX1^TAP^ resulted in the same set of interactors as purification of SS18^TAP^, with the exception of peptides originating from the SSX1 moiety of the oncofusion protein (Table 1). All subunits of the SWI/SNF BAF variant complex were identified, as well as the novel interactors GLTSCR1, DPF2 and its paralog DPF1, BRD9, and BCL7A and its paralogs BCL7B and C (Table 1, Figure 1B). We conclude that, similarly to SS18, the SS18-SSX1 oncofusion protein also resides in both the ARID1A and ARID1B-bearing BAF variants of human SWI/SNF.

### DPF2 resides in BAF

**Figure 2:**
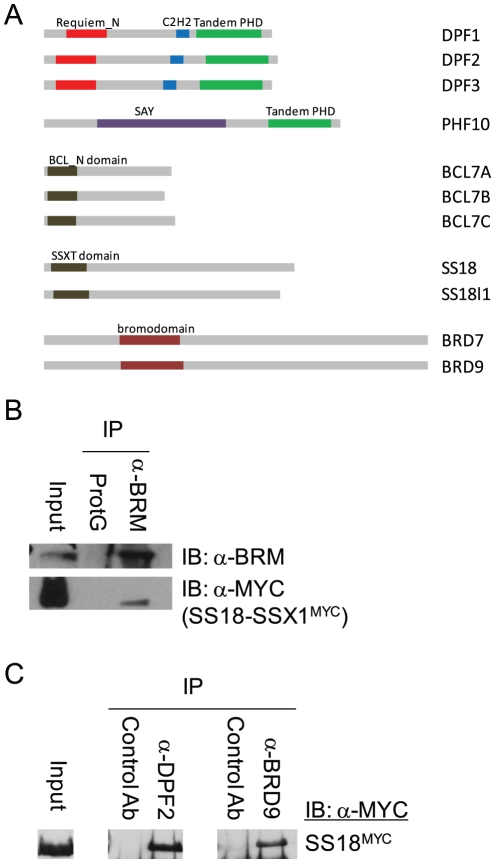
SS18 and the animal-specific SWI/SNF subunits. (A) Domain organization of the human proteins DPF1,-2,-3; PHF10; SS18 and its paralog SS18l1/crest; and BCL7A,-B,-C. We note that while CG2682, the *Drosophila melanogaster* ortholog of DPF2 (Table 2), lacks the C2H2 domain, this domain is present in the *Tribolium* ortholog D6WFQ9_TRICA [125], [132], [133], suggesting conservation of this domain in protostome and in deuterostome animals (B) Co-immunoprecipitation of SS18-SSX1^MYC^ by antibodies directed against human BRM. (C) Co-immunoprecipitation of SS18^MYC^ by antibodies directed against DPF2 and BRD9.

DPF2, also known as ubi-d4 or Requiem, is ubiquitously expressed and implicated in apoptosis [92]. It belongs to the d4 family which in humans consists of three paralogous genes: neuro-d4 (DPF1), ubi-d4 (DPF2) and cer-d4 (DPF3) [98], [99]. This gene family is not present in any of the currently sequenced plant genomes. Figure 2A shows that DPF factors harbor a conserved N-terminal domain (Pfam14051, [100]), a central C2H2-type Krüppel zinc finger motif with potential nucleic acid binding activity and C-terminal double paired finger PHD domains that have been shown to mediate conditional protein-protein interactions [42], [101], [102]. DPF1, 2, 3 and PHF10 were named BAF45A-D [41] because they were found in biochemical SWI/SNF preparations.

The DPF2^TAP^ purification results indicate that DPF2 resides mainly in ARID1-bearing BAF complexes, since no polybromo or ARID2 peptides were identified, whilst high confidence ARID1A and ARID1B peptides were detected (Table 1). Furthermore, like SS18^TAP^, DPF2^TAP^ co-purified BCL7A and BRD9 as well as the SS18 paralog, SS18l1. Association of DPF2 with SS18 was further confirmed by co-immunoprecipitation (Figure 2C).

### PHF10 resides in PBAF

PHF10 harbors two PHD domains but it is not a member of the DPF paralog group as it lacks a central Krüppel zinc finger motif, and harbors a SAY domain that is conserved in animals but not plants [103] (Figure 2A). In contrast to DPF2^TAP^, PHF10^TAP^ was second only to INI1^TAP^ in its yield of the PBAF-specific subunits ARID2 and polybromo (Table 1), demonstrating a strong association with PBAF-class SWI/SNF complexes. None of the BAF-associated SS18, DPF or BCL7 factors were detected in PHF10^TAP^ preparations, suggesting that PHF10 indeed resides in a distinct subset of SWI/SNF complexes. Complete exclusion of PHF10 from BAF complexes may not be the case however, since one high confidence ARID1A-derived peptide was identified. Whether this reflects physiological subunit exchange between subtypes of SWI/SNF complexes or mal-assembled complexes remains an open question.

Notably, high confidence BRD7 peptides were detected, similar to the INI1^TAP^ purification (Table 1), suggesting that PHF10 forms PBAF-type SWI/SNF complexes that can harbor BRD7 but not BRD9 since, PHF10^TAP^ did not pull down BRD9, in contrast to SS18^TAP^, SS18-SSX1^TAP^ and BCL7C^TAP^ which did pull down BRD9 (Table 1, Figure 1B).

### BCL7 proteins reside mainly in BAF

Similar to multiple SWI/SNF subunits, BCL7 family members have been implicated in carcinogenesis [93], [94]. The presence of BCL7 family members in SWI/SNF complexes has been reported before [43]. We succeeded in purifying BCL7A^TAP^ and BCL7C^TAP^-associated proteins (Table 1, Figure 1B). In both cases and in contrast to INI1^TAP^ and PHF10^TAP^, next to the core SWI/SNF subunits we also recovered more BAF-specific ARID1A and ARID1B subunit peptides (Table 1, Figure 1B), suggesting that BCL7 factors are mainly subunits of BAF complexes. The comparatively low levels of the PBAF-specific subunits in the BCL7C^TAP^ preparations may be due to the high levels of BCL7C^TAP^ in the cell line that was employed (Figure 1A) and indicate that the distinction between BAF and PBAF complexes by BAF subunits can be blurred operationally (Figure 1B). The absence of the PBAF-specific SWI/SNF subunit PHF10 from both BCL7^TAP^ purifications strengthens the notion that BCL7 factors mainly associate with the BAF variants of SWI/SNF, however. Furthermore, the fact that BCL7C^TAP^ co-purified DPF2, like BCL7A^TAP^, as well as the DPF2 paralogs DPF1 and DPF3 suggest that these two DPF2 paralogs also associate with BAF complexes. Indeed, co-purification of DPF1 and DPF3 parallels the purification results obtained with SS18-SSX1^TAP^ (see above) again pointing towards the BAF variants of SWI/SNF. Finally, like DPF2^TAP^, BCL7C^TAP^ also pulled down SS18l1 (Table 1, Figure 1B), a paralog of SS18 also known as CREST which has previously been linked physically to ARID1B [104], again strengthening the conclusion that BCL7A and BCL7C are mainly subunits of the BAF variants of SWI/SNF complexes. Interestingly, orthologs of BCL7 can only be found in sequenced animal genomes.

### BRD9 associates with BAF

A well established function of bromodomains is to recognize specific acetylated lysines. The paralogous catalytic subunits of SWI/SNF, BRG1 and BRM harbor one C-terminal bromodomain that is closely related to the six bromodomains of polybromo, but quite distinct from the bromodomains of BRD7 and BRD9 [105]–[107].

A FLAG-BRD7 fusion has been reported to purify PBAF complexes [43]. We were not able to successfully perform BRD7^TAP^ purifications (data not shown). However, BRD9^TAP^ did yield significant mass spectrometry results (Table 1, Figure 1). BRD9^TAP^ yielded peptide hits for at least one paralog of each core SWI/SNF subunit and, contrary to what was reported for BRD7 [43], [108], the presence of high confidence ARID1A and ARID1B peptides indicates inclusion of BRD9 in BAF complexes. This notion is buttressed by the presence of SS18 and BCL7C amongst the proteins co-purifying with BRD9^TAP^ (Table 1, Figure 1, Data S1). Association of BRD9 with SS18^TAP^ was further confirmed by co-immunoprecipitation (Figure 2C).

We quantified our mass spectrometry data on the basis of the exponentially modified protein abundance index (emPAI, Table 1, Figure 1) and this revealed that BRD9^TAP^ did not efficiently purify SWI/SNF (Table 1), in keeping with our gel electrophoresis analysis (Figure 1A). The major factors we identified in our BRD9 preparation are the DEAD box ATP-dependent RNA helicases DDX3X, DDX5 and DDX17 and the RNA binding factor RBM14/COAA (Table 1). Since Emerson and co-workers reported substantially higher ATPase activity in their BRD7 preparations than predicted by BRG1/BRM content, it may perhaps be that BRD7 also co-purifies the DEAD box RNA helicases DDX3X, DDX5 and/or DDX17 [43], [109], [110]. Similarly, RBM14/COAA is a nuclear receptor co-activator [111]. Furthermore, RBM14/COAA has previously been reported to associate with SS18 in yeast two hybrid assays [112], [113]. However, arguing against a direct interaction between SS18 and RBM14, we did not detect RBM14/COAA when SS18^TAP^ or SS18-SSX1^TAP^ associated factors were purified (Table 1, Figure 1B).

### Putative BAF associated proteins

Crabtree and colleagues [49] published a list of putative novel BAF-associated proteins which we have monitored in this data set. Hence, we also detected GLTSCR1 in our SWI/SNF complex preparations (Table 1). GLTSCR1 is a candidate tumor suppressor gene for gliomas [114]. As we detected GLTSCR1 in four of five BAF purifications (Table 1), our results support the notion that GLTSCR1 is a BAF-associated factor, but this will need to be confirmed directly.

Of the other putative novel BAF-associated proteins, we could detect NONO and its binding partner SFPQ [115], [116], however, at levels that were not much higher than in control purifications (Table 1). Thus, although our data do not exclude an interaction with SWI/SNF, more experimental evidence is needed on this front. Finally, the proposed putative BAF-associated factors NUMA1, SRRM2 and MYBBP1A [49] were not detected in any of our SWI/SNF purifications (Table 1), suggesting weak biochemical association with SWI/SNF in the ‘293’ human embryonic kidney cell line, if any.

## Discussion

Paralogous human SWI/SNF subunits are known to be expressed in tissue and signal specific fashion, generating alternative SWI/SNF complex configurations that can cooperate with transcription factor networks to coordinate cell proliferation and differentiation. Here, we focus on SWI/SNF subunits that are absent from yeast but conserved in animals and plants (SS18) or only in animals (DPF, BCL7 and PHF10) (Table 2).

Essentially there are two types of human SWI/SNF complexes [32], [44]; those that harbor the polybromo/BAF180 and ARID2/BAF200 subunits (PBAF-class) and those that harbor either ARID1A/BAF250a or ARID1B/BAF250b (BAF-class) [117]. A similar bi-partition exists in *Drosophila melanogaster* except that there is only one ARID1 ortholog, namely OSA [118], [119]. Similarly, the fly genome only encodes one ortholog of the mammalian SMARCC (CG18740/moira) and of BRD7/9 (CG7154), SS18 (CG10555), DPF2 (CG2682/d4) and BCL7 (CG17252/BCL7-like) protein coding genes (Table 2).

Our mass spectrometry analysis of affinity tag-mediated protein complex purifications confirms bipartition of SWI/SNF complexes in BAF and PBAF-class complexes. We demonstrate here that the paralogous cancer-related minor SWI/SNF subunits DPF1, -2, -3; BCL7A, -B, -C; and SS18 and SS18L1 reside in BAF-class human SWI/SNF complexes and, that PHF10 marks PBAF SWI/SNF complexes. Moreover, because quantitative analysis indicates that the chief interaction partners of PHF10, DPF2, SS18, SS18-SSX1, BCL7A and BCL7C are the other SWI/SNF subunits, we speculate that they exert their molecular action through their respective SWI/SNF complexes. It remains to be seen indeed to what extent our results, which were obtained in one human cell line, can be extrapolated to other cell types and even other organisms. Considering the congruence between our data and a recent studies on *Drosophila* SAYP [120] and a large scale proteomic survey of nuclear receptor co-activators [121], we believe they can. Moreover, there may be more as yet ‘undiscovered’ human SWI/SNF subunits, such as the putative GLTSCR1 subunit [49], and these may also be present in our data sets (Data S1), which can be mined by interested investigators.

Since we could not detect notable differences between SS18 and its oncogenic fusion products at the proteomic level, the oncogenic activity of the SS18-SSX fusions may have to be sought either at the level of SWI/SNF (dis)assembly dynamics, post-translational modifications or an affinity for specific genomic loci [42], [106], [122], although it is formally possible that the SS18-SSX oncofusion proteins undergo different proteomic interactions in the elusive synovial sarcoma precursor cell type than those we detected in Hek293 cells [123].

Interestingly, we detected the bromodomain proteins BRD7 and BRD9 in our SWI/SNF preparations. The fly protein CG7154 is that organism's sole BRD7/BRD9 ortholog. It will be interesting to determine whether it associates with the fly brahma SNF2 ATPase, and if so, whether it is specific to the BAP or PBAP fly equivalents of BAF and PBAF. In humans, BRD7 appears to promote cellular senescence [108], [124], and, in line with our results, it has convincingly been linked to the PBAF complex [43]. On the other hand, our data make a novel link between BRD9 and the BAF complex defined by SS18, DPF and BCL7, since we recovered it when BCL7C^TAP^, SS18^TAP^ and the SS18-SSX1^TAP^ were used as affinity bait and since in the reciprocal experiment, BRD9^TAP^ purifications contained SS18 and BCL7A. We note however that BRD9^TAP^ did not efficiently pull down BAF proteins in our experimental set-up (Figure 1A). Whether this reflects a status as a minor though BAF-specific subunit or a technical limitation is an open question.

Altogether, this work demonstrates that paralogs of SS18, BCL7 and DPF factors, which can be found in both protostome [125]) and deuterostome animals (Table 2), together define a novel class of BAF-type SWI/SNF complexes that is restricted to the animal lineage. Finally, our data indicate that PHF10 versus DPF1, -2, -3 respectively mark PBAF versus BAF-type SWI/SNF complexes in a mutually exclusive fashion.

## Methods

### Constructs

Tandem Affinity Purification (TAP) constructs were generated by PCR using the oligomers indicated in parentheses and cloned into the XhoI and EcoRI restriction sites in the retroviral expression vector pZXN, whereby the TAP-tag sequence was fused to the coding sequences at their N-terminus [91]. SS18 (isoform 2, cgtactGAATTCATGTCTGTGGCTTTCGCGG, tgacttCTCGAGTCACTGCTGGTAATTTCCATACT) and SS18-SSX1 (cgtactGAATTCATGTCTGTGGCTTTCGCGG, tgtcatCTCGAGTTACTCGTCATCTTCCTCAGGGT) coding sequence were amplified by PCR from pIRES2 vectors [126]. DPF2 (cgtatcGAATTCATGGCGGCTGTGGTGGAGAAT, tgtcttCTCGAGTCAAGAGGAGTTCTGGTTCTGGTA), BCL7C (cgtatcGAATTCATGGCCGGCCGGACTGTA, tgtcttCTCGAGTCAGGGGTCAGGGGCATTT), BRD9 (atacttGAATTCATGAAGGGATACCAAAGTCTTGTATTC, tactatCTCGAG TTAGGTCTTGGCAGAGGCCGCA) and PHF10 (gaattcGAATTCATGCTTCAAGAACAAGTCAGTG, aagcttAAGCTTTTATCCCTCTTTGCTGTTTTTCC, cloned into pBSIISK+ cut by the same enzymes and then released using SalI and EcoRI and further subcloned) coding sequences were amplified from cDNA clones (RZPD or OriGene). BCL7A (isoform 2, ttacttCAATTGATGTCGGGCAGGTCGGGT, tacttaGTCGACCTACATCTCTTCGGAGTTTTGTTG) was amplified from cDNA of Hek293 cells. INI1 (atacttGAATTCATGAAGGGATACCAAAGTCTTGTATTC, tactatCTCGAGTTAGGTCTTGGCAGAGGCCGCA) was amplified from pUHD-10-3-INI1 [127]. The retroviral vector pZXN is derived from the pZOME-1N vector (Euroscarf) and contains a TAP-tag consisting of one protein A domain followed by two tobacco etch virus cleavage sites (TEV) and then either a MYC epitope (GCCGGCAAGCCCCGGCATATGAATTTAATGGAGCAGAAGCT TATCAGCGAGGAGGACCTGGGCGGGGAATTC) or, in the case of PHF10, a TY1 epitope (GCCGGCGCCGATGCCGGCAAGCCCCGGCA TAGGACCGGTGAGGTGCACACCAACCAGGACCCCCTGGACGAATTC) 5′ of the EcoRI cloning site. Every clone was verified by DNA sequencing.

### Cell culture and stable cell lines

Human Embryonic Kidney (Hek293, ATCC CRL-1573) and phoenix cells were grown in Dulbecco's modified Eagles medium (Invitrogen) supplemented with 10% FCS, penicillin 100 µg/ml and streptomycin 100 U/ml (Invitrogen) at 37°C in 5% CO_2_. Retroviral stable cell lines were generated as previously described [91]. Briefly, phoenix amphotropic packaging cells were transfected with 20 µg retroviral plasmid pZXN-SS18, pZXN- SS18-SSX1, pZXN- SS18-SSX2, pZXN-DPF2, pZXN-BRD9, pZXN-BCL7A, pZXN-BCL7C, pZXN-PHF10 or pZXN-INI1 after which Hek293 cells were transduced with virus containing supernatant in two infectious rounds of 24 hours in the presence of 8 µg/ml polybrene. Clones were selected with 1 µg/ml puromycin and tested for recombinant protein expression. Transduction of SS18 and SS18-SSX TAPtag fusions in the syo-1 synovial sarcoma cell line [128] were not successful (data not shown). Since Hek293 cells expressed the transduced transgenes efficiently and could be expanded as desired, we performed our study with this cell line.

### Tandem Affinity Purification

Tandem Affinity Purification was performed as previously described in detail [91]. Shortly, whole cell extracts from cell lines expressing TAP-tagged proteins were incubated with IgG sepharose beads (Pharmacia). After TEV cleavage the TEV eluates were pre-cleared with protein A beads and used for immunoprecipitation with anti-MYC or anti TY1 epitope antibodies. Proteins were eluted from the beads by peptide elution, loaded on a SDS-PAGE gel and visualized by silver staining. The same protocol was employed for all the purifications reported here.

### Co-immunoprecipitation

Co-immunoprecipitations were performed on TEV cleavage eluates obtained as described above, using antibodies directed against BRM (Abcam 15597), DPF2 (Aviva systems biology ARP33221_P050) or BRD9 (Aviva systems biology ARP34803_T200), under the same conditions as the anti-MYC or TY1 immunoprecipitations in the TAPtag purification protocol. The immunoprecipitated proteins were separated by SDS-PAGE, transferred onto nylon filters and probed with anti-MYC antibodies, which recognize the transduced SS18-SSX1 (Figure 2B) or SS18 (Figure 2C) proteins.

### Mass spectrometry

The silver stained gel lanes were cut into small pieces. After reduction and alkylation the proteins were trypsin (Promega) digested and extracted from the gel using trifluoroacetic acid (TFA). Peptides were sequenced using a nano-high-pressure liquid chromatography Agilent 1100 nanoflow system connected online to a 7-Tesla linear quadrupole ion-trap Fourier transform (FT) mass spectrometer (Thermo Electron, Bremen, Germany) essentially as described previously [129]. MSquant software package (http://www.msquant.sourceforge.net) was used to parse the raw files and for generation of peak lists. The mascot algorithm was used to identify the proteins [130]. Exponentially Modified Protein Abundance Index (emPAI) factors were calculated as described previously [96], using high confidence peptides (Data S1, MASCOT score≥20, delta≥5, error≤5; 400–6000 Da).

### Expression profiling

Expression profiling was performed on four Hek293 polyA mRNA samples by microarray analysis using Affymetrix human exon array 1.0 ST according to manufacturer instructions (Data S2).

## Supporting Information

Data S1 Mass spectrometry results, including; accession numbers, short protein descriptions, peptide sequences, associated Mascot score, peptide delta score and absolute calibrated mass relative error.
(XLS)

Data S2 Quadruplate polyA mRNA expression profile of Hek293 cells determined with the Affymetrix human exon array 1.0 ST platform.
(XLS)
